# Laquinimod treatment in the R6/2 mouse model

**DOI:** 10.1038/s41598-017-04990-1

**Published:** 2017-07-10

**Authors:** Gisa Ellrichmann, Alina Blusch, Oluwaseun Fatoba, Janine Brunner, Christiane Reick, Liat Hayardeny, Michael Hayden, Dominik Sehr, Konstanze F. Winklhofer, Carsten Saft, Ralf Gold

**Affiliations:** 1 Department of Neurology, St. Josef-Hospital, Ruhr-University Bochum, Bochum, Germany; 2 Center of Clinical Research, Ruhr-University Bochum, Bochum, Germany; 3 Galmed Pharmaceuticals, Tel Aviv, Israel; 4 Teva Pharmaceutical Industries Ltd, Tiqva, Israel; 5 Department of Molecular Cell Biology, Institute of Biochemistry and Pathobiochemistry, Ruhr-University Bochum, Bochum, Germany

## Abstract

The transgenic mouse model R6/2 exhibits Huntington’s disease (HD)-like deficits and basic pathophysiological similarities. We also used the pheochromocytoma-12 (PC12)-cell-line-model to investigate the effect of laquinimod on metabolic activity. Laquinimod is an orally administered immunomodulatory substance currently under development for the treatment of multiple sclerosis (MS) and HD. As an essential effect, increased levels of BDNF were observed. Therefore, we investigated the therapeutic efficacy of laquinimod in the R6/2 model, focusing on its neuroprotective capacity. Weight course and survival were not influenced by laquinimod. Neither were any metabolic effects seen in an inducible PC12-cell-line model of HD. As a positive effect, motor functions of R6/2 mice at the age of 12 weeks significantly improved. Preservation of morphologically intact neurons was found after treatment in the striatum, as revealed by NeuN, DARPP-32, and ubiquitin. Biochemical analysis showed a significant increase in the brain-derived neurotrophic factor (BDNF) level in striatal but not in cortical neurons. The number of mutant huntingtin (mhtt) and inducible nitric oxide synthase (iNOS) positive cells was reduced in both the striatum and motor cortex following treatment. These findings suggest that laquinimod could provide a mild effect on motor function and striatal histopathology, but not on survival. Besides influences on the immune system, influence on BDNF-dependent pathways in HD are discussed.

## Introduction

HD is a rare but fatal autosomal dominantly inherited neurodegenerative disorder that is characterized by motor dysfunction, cognitive decline, and emotional as well as psychiatric symptoms^1^. The responsible mutation, an abnormal expansion of a CAG codon ≥36 repeats, is located in exon 1 of the huntingtin gene (HTT) on chromosome 4. The expansion encodes a prolonged polyglutamine (polyQ) sequence that results in conformational change of the huntingtin (htt) protein and, therefore, induces the formation of intranuclear inclusions of mhtt in the brain. These are pathogenic for HD and can be found in both patients and R6/2 mice (141–157 CAG repeats) as an experimental model of HD^2^. The striatum is the most affected area, with progressive atrophy accompanied by neuronal cell loss^3^. Underlying mechanisms are still not known in detail: mitochondrial dysfunction, oxidative stress, reduced amounts of trophic factors like brain-derived neurotrophic factor (BDNF) as well as inflammatory processes have been implicated^4–9^.

BDNF is presumably produced by cortical neurons and delivered to the striatum via cortico-striatal anterograde transport^10, 11^. It is very important for neuronal survival. The most important projection neurons of the striatum are the medium spiny neurons (MSNs). MSNs express TrkB that mediates BDNF neurotrophic support of MSNs as they are probably not able to produce BDNF by themselves^12^. For survival and maintenance they rely on cortical, thalamic, and midbrain BDNF support that depends on the BDNF-TrkB signaling pathway^10, 13–15^. Lack of BDNF, therefore, induces early degeneration of MSNs, which is a central pathomechanism in HD.

BDNF also controls glutamate release and protects neurons from degeneration^16–18^. There are conflicting studies, if BDNF is decreased in the brains of HD patients and animal models for HD^19, 20^. In contrast to other neurodegenerative diseases, only in HD was BDNF found to be linked mechanistically to the underlying genetic mutation^21^. Wild-type htt seems to act as a regulator of the neuron-restrictive silencer element (NRSE) and thus the transcription of NRSE regulates genes via the repressor element-1 transcription factor/ neuron-restrictive silencer factor (REST/NRSF). The regulation is impaired with mhtt^22^. Therefore, promoting BDNF levels might be a potential therapeutic target in this devastating disease for which there is, to date, no known cure.

Laquinimod is a quinoline-3-carboxamide derivate (laboratory code: ABR-215062) with linomide (roquinimex) as the lead compound. Originally, it was an orally immunomodulatory substance developed for the treatment of relapsing-remitting MS with striking properties concerning the reduction of brain atrophy^23, 24^. Trials in HD are ongoing (LEGATO-HD, phase II). Its oral bioavailability is described as approximately 80–90% with low plasma protein binding. After metabolization in the liver by the cytochrome isoenzyme CYP3A4, laquinimod is eliminated in the urine. Laquinimod is known to cross the blood-brain barrier^25, 26^. Increased levels of BDNF and anti-apoptotic effects were observed after laquinimod treatment and may, therefore, account for a neuroprotective capacity of this drug^27, 28^.

The therapeutic potential of laquinimod was already seen in the YAC128 mouse model of HD regarding motor function, behavior, and histopathology^29^.

Here, we explore the potential of laquinimod in the R6/2 transgenic mouse model of HD. R6/2 mice mimic many histopathological aspects of HD. They transgenically express the exon 1 of the human HD gene with 155–165 CAG repeats^30^. Exon 1 of the HD gene with an expanded CAG repeat is sufficient to cause a progressive neurodegenerative phenotype in transgenic mice^30^.

In R6/2 mice, motor symptoms like dyskinesia, ataxia, clasping behavior, epileptic seizures, and spontaneous shivering movements start at about six weeks of age. From the age of nine-ten weeks, there is a significant neuronal dysfunction and mice display neuronal atrophy in the striatum^31^. Continuous weight loss leads to death between 11 and 14 weeks of age.

The pheochromocytoma-12 (PC12)-cell-line-model we used to investigate the effect of laquinimod on metabolic activity consists of clonal cells originated from a transplantable rat pheochromocytoma. As a cell-line-model in HD, they have a construct of complete exon1 and 103 repeats fused to enhanced green fluorescent protein (EGFP) as reporters (PC12-mhtt-exon 1-103QP-EGFP). PC12 cells are stably transfected with a hybrid ecdysone receptor. The cells respond to nerve growth factor (NGF) by the change of phenotype and neurite growth^32^.

## Results

### Laquinimod and its effects on body weight and survival in R6/2 mice

**Figure 1:**
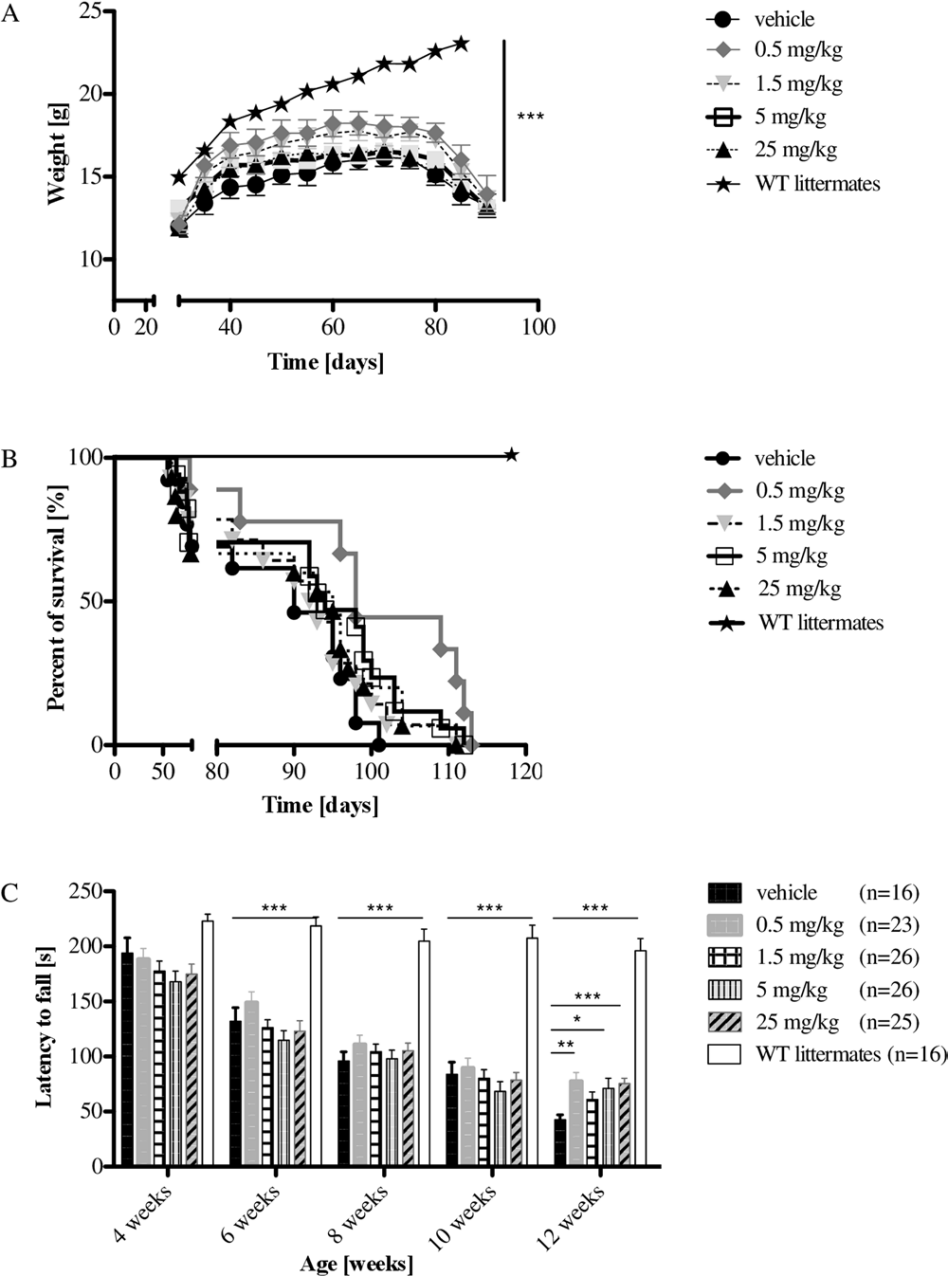
Weight analysis, survival, and motor function of R6/2 mice. (**A**) R6/2 mice were treated with four different concentrations of laquinimod and weight course was compared to vehicle treated R6/2 mice and WT littermates as control. Treatment with lower concentrations of laquinimod (0.5 mg/kg =  and 1.5 mg/kg = ) prevented weight loss. There was no significant difference. n-number: vehicle 16 ●, 0.5 mg/kg 23  , 1.5 mg/kg 26  , 5 mg/kg 26 □, 25 mg/kg 25 ▲, WT littermates 16 *. (**B**) Kaplan-Meier survival analysis of R6/2 mice after treatment with laquinimod further strengthened the trend of weight course and confirmed, that application of 0.5 mg/kg laquinimod seems to prolong survival in R6/2 mice. Again, there was no significant difference. n-number: vehicle 13 ●, 0.5 mg/kg 10  , 1.5 mg/kg 14  , 5 mg/kg 17 □, 25 mg/kg 15 ▲, WT littermates 10 *. (**C**) Rotarod analysis in R6/2 mice at different time point [weeks]. As characteristic of R6/2 mice, all groups worsened in motor function during lifespan; this is reflected in total latency to fall which is reduced from a maximum of about 200 sec in week 4 to about 120 sec in week 8 and only 85 sec in week 12. In comparison to the vehicle, all laquinimod-treated groups achieved better results at the latest time point. There was a significant difference with p-value of p* < 0.05 in 5 mg/kg group, p** < 0.01 in 0.5 mg/kg group, p*** < 0.001 in 25 mg/kg group) compared to vehicle. n-number: vehicle 16, 0.5 mg/kg 23, 1.5 mg/kg 26, 5 mg/kg 26, 25 mg/kg 25, WT littermates 16.

R6/2 mice aggressively and rapidly manifest HD-like symptoms and, generally after a permanent increase of weight during their growth period, they lose body weight from approximately at eight-nine weeks of age^30^. The loss of body weight in treatment groups and controls was concurrent with progressive motor deficits. However, there was no effect of laquinimod on body weight in R6/2 mice (Fig. 1A).

Median survival was 86.6 days (SD 13.4, SEM ± 3.7) in the vehicle group and 99.8 days (SD 12.8, SEM ± 4.3) after treatment with laquinimod 0.5 mg/kg (p-value = 0.1) (vehicle: n = 13, 0.5 mg/kg: n = 10, 1.5: n = 14, 5: n = 17, 25: n = 15). Again, these results were not statistically significant in a Kaplan-Meier analysis (Fig. 1B). Various n-numbers are accounted for by using animals for histochemical and immunohistochemical analyses at selected time points during the progressive disease course.

### Laquinimod improved motor coordination and balance in older R6/2 mice

Characteristically, R6/2 mice develop motor impairment during their lifespan, beginning parallel to weight loss at the age of eight-nine weeks. Upon analysis of motor performance, latency-to-fall values during rotarod testing in the complete R6/2 mice cohort were not significantly different at baseline (week 4) and up to week 10 (Fig. 1C). However, at the age of 12 weeks, 0.5, 5, and 25 mg/kg treated R6/2 mice remained on the rotarod for a significantly longer period of time than controls (0.5 mg: p** < 0.01; 5 mg: p* < 0.05; 25 mg: p*** < 0.001) (Fig. 1C).

### Effects of laquinimod on neuronal survival and aggregates

**Figure 2:**
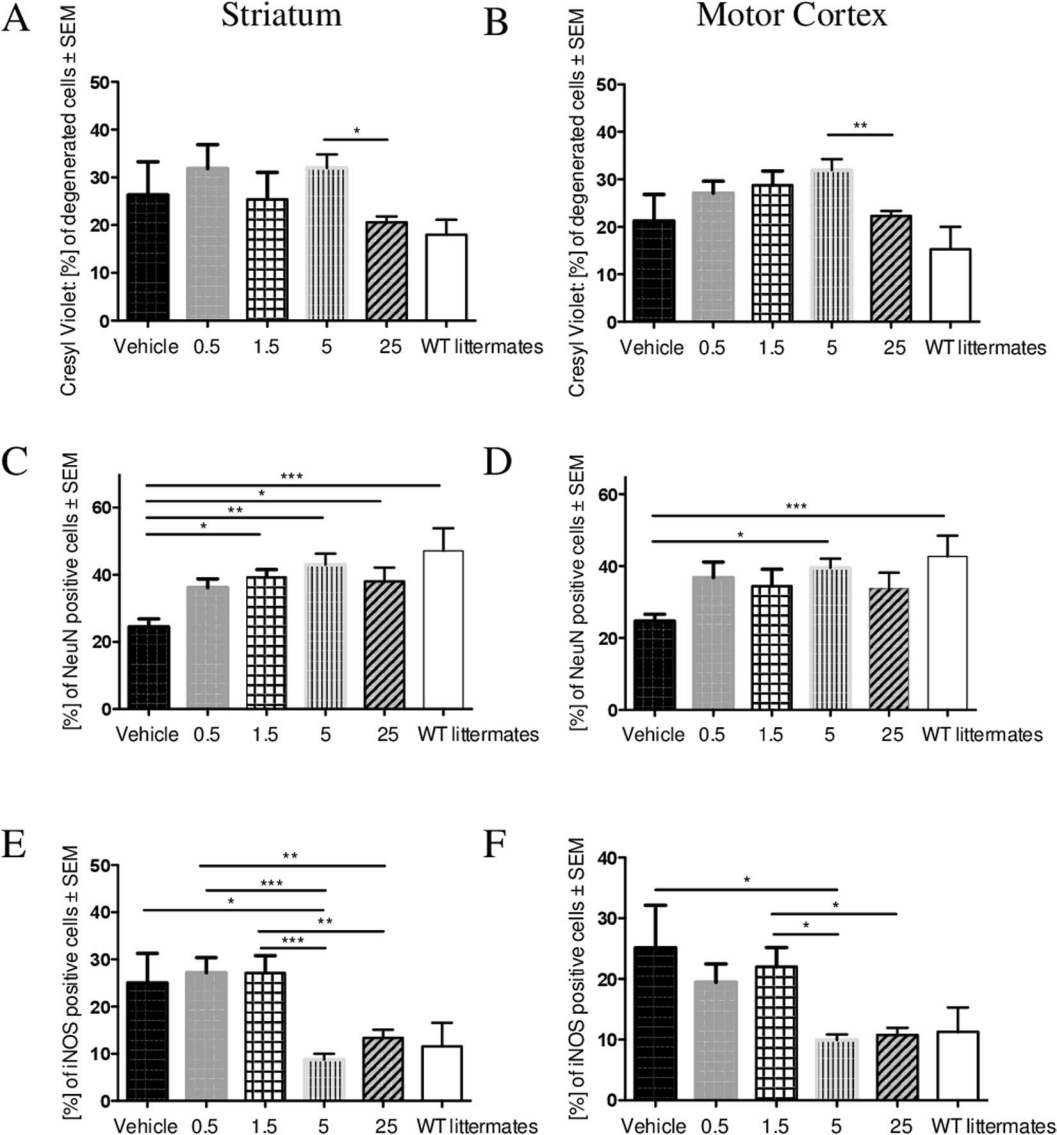
Effects of laquinimod on neuronal pathology in R6/2 mice. We analyzed striatum (**A**,**C**,**E**) and motor cortex region (**B**,**D**,**F**) of R6/2 mice after different stainings. Note the pronounced neuronal pathology and neuronal loss in this advanced stage of disease (**A**–**D**). After cresyl violet staining (**A**,**B**) stereological quantification did neither demonstrate a significant preservation of neuronal cell numbers in the striatum nor in the motor cortex of laquinimod-treated R6/2 mice in comparison to vehicle group (vehicle: n = 3, 0.5 mg: n = 3, 1.5 mg: n = 3, 5 mg: n = 7, 25 mg: n = 7, WT littermates: n = 6). Laquinimod treatment, especially 5 mg/kg dosage, reduced NeuN-positive cell loss in R6/2 striata and motor cortices (**C**,**D**). Blinded quantification of iNOS positive cells led to increased levels both in the striatum and the motor cortex with 5 mg/kg laquinimod being most effective (**E**,**F**) (vehicle: n = 3, 0.5 mg: n = 6, 1.5 mg: n = 7, 5 mg: n = 5, 25 mg: n = 7, WT littermates: n = 6).

Next, we were interested in the effects of laquinimod treatment on CNS morphology in our HD mouse models, and performed cresyl violet staining (Bregma 0.14 mm to Bregma 1.10 mm) to quantify neuronal pathology in a blinded manner (Fig. 2A). In R6/2 mice, quantification was performed at day 80-84 with a group size of two to seven (vehicle: n = 2, 0.5 mg/kg: n = 5, 1.5: n = 4, 5: n = 7, 25: n = 7). At this advanced disease stage, a marked neuronal pathology is common in the motor cortex as well as in the striatum. Compared to the vehicle group and WT littermates laquinimod-treated R6/2 mice did not show a significant preservation of neuronal cell numbers in neither the striatum nor the motor cortex (Fig. 2A,B).

As cresyl violet can stain glia as well, we additionally used the more specific marker NeuN (Fig. 2C,D). There was less neuronal loss after laquinimod treatment in both striatum and motor cortex, with a dosage of 5 mg/kg being most effective (p** < 0.01). N-number was similar to cresyl violet staining.

In our study, laquinimod treatment of R6/2 mice led to decreased levels of the NOS subtype inducible NOS (iNOS) (Fig. 2E,F) both in the striatum and the motor cortex with a maximal effect in 5 mg/kg group (p* < 0.05; vehicle: n = 2, 0.5: n = 5, 1.5: n = 4, 5: n = 7, 25: n = 7). The rationale for the quantification of iNOS positive cells was to see if there are any changes in activated microglia after laquinimod treatment. We consciously decided to use a more general staining to get an idea of the effect. Additional and more specific stainings for microglial markers are in preparation in a separate project.

**Figure 3:**
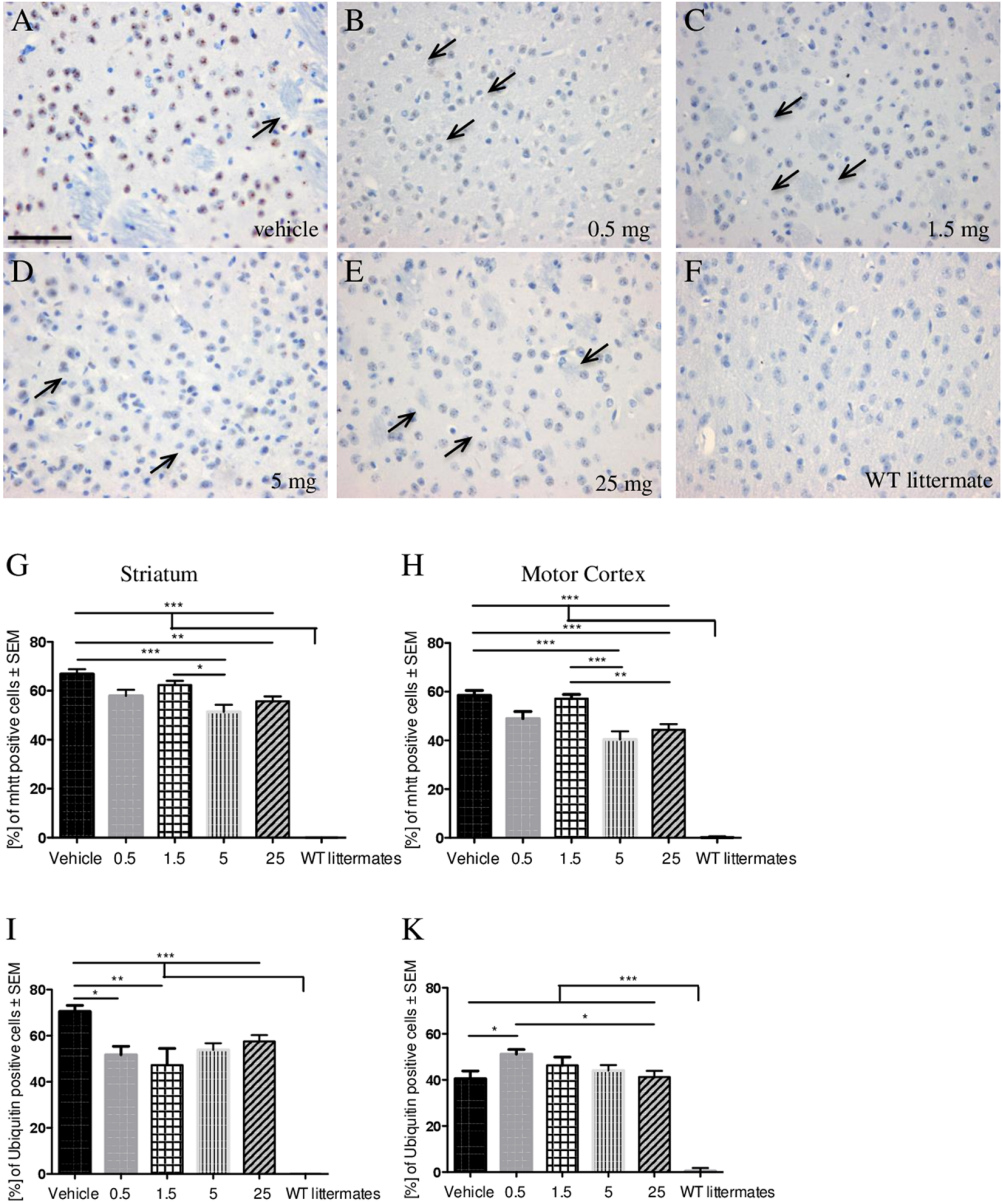
Effect of laquinimod on htt aggregates. Focusing htt aggregates we used direct htt staining (**A**–**H**) (vehicle: n = 5, 0.5 mg: n = 3, 1.5 mg: n = 4, 5 mg: n = 7, 25 mg: n = 8, WT littermates: n = 6) and ubiquitin staining (**I**,**K**) (vehicle: n = 4, 0.5 mg: n = 7, 1.5 mg: n = 5, 5 mg: n = 5, 25 mg: n = 6, WT littermates: n = 6). There were significant differences of htt inclusions with reduction of inclusions after treatment and 5 mg/kg laquinimod being most effective. Mainly in striatum area, a similar trend was seen after ubiquitin staining. Analysis revealed a significantly lower number of ubiquitin-positive cells (p* < 0.05) in the 0.5 mg-laquinimod-group in both areas (**I,K**).

In a subsequent step, we analyzed whether a beneficial effect of laquinimod in R6/2 mice might be associated with a decrease in htt aggregation. Both after staining for htt aggregates with the EM48 antibody, which has a high affinity for mhtt (Fig. 3A–H), and ubiquitin as an indirect marker for protein aggregates (Fig. 3I,K), there were significant differences of mhtt inclusions after treatment with laquinimod. All laquinimod groups (0.5: n = 5, 1.5: n = 4, 5: n = 6, 25: n = 8) showed a reduced percentage of mhtt positive cells compared to the vehicle (vehicle: n = 3) with 5 mg/kg laquinimod being the most effective concerning reduced htt aggregation (p*** < 0.001) (Fig. 3G,H). A similar result was seen after ubiquitin staining focusing on the striatum (Fig. 3I). Here, concentrations of 0.5 and 1.5 mg/kg led to the lowest levels of ubiquitin-positive cells (vehicle: n = 4, 0.5: n = 7, 1.5: n = 5, 5: n = 4, 25: n = 4; p* < 0.05). In contrast, levels of ubiquitin-positive cells slightly increased after treatment with 0.5 mg/kg in the motor cortex (p* < 0.05) (Fig. 3K).

**Figure 4:**
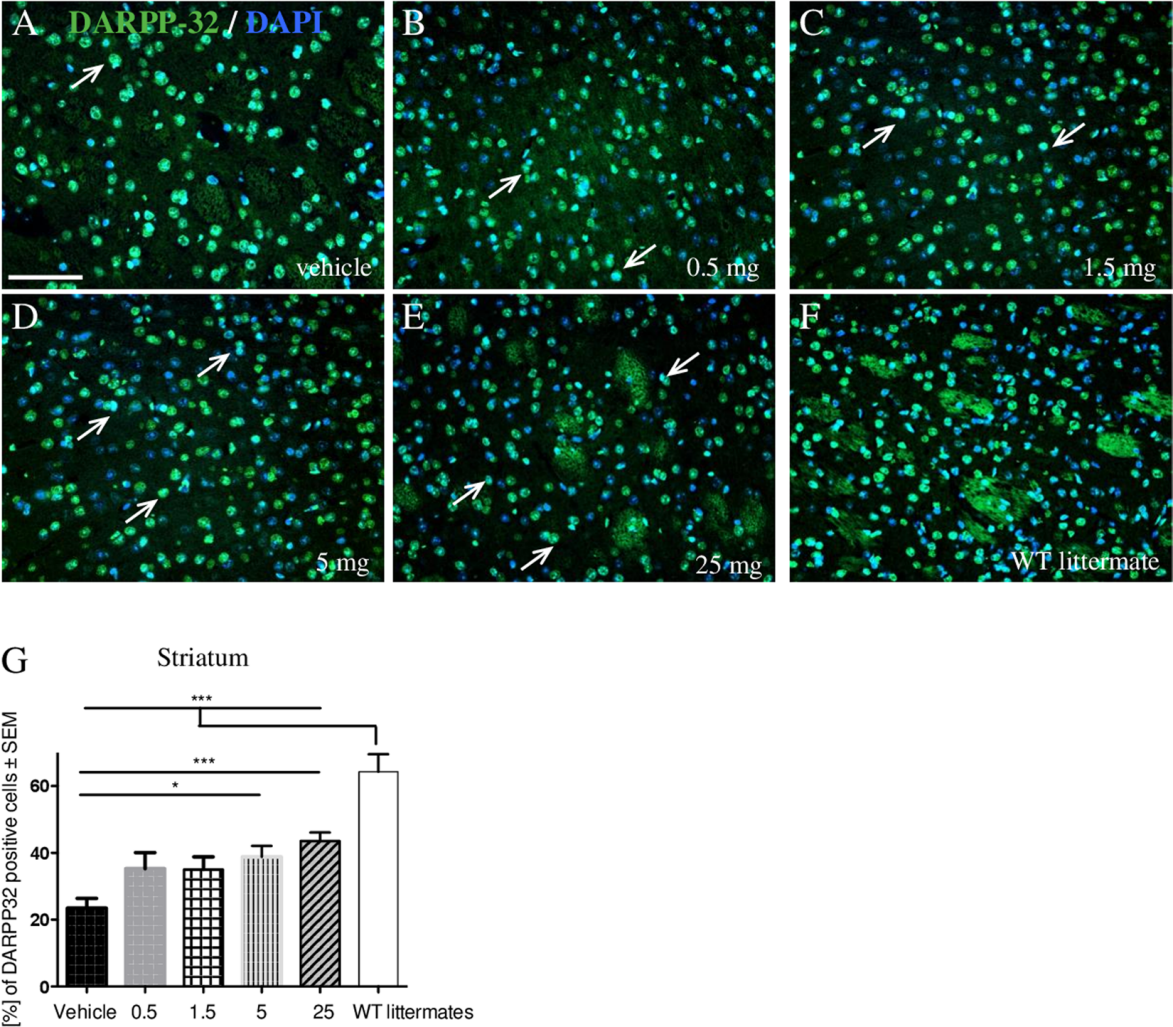
Analysis of neuroprotective effects in DARPP-32 staining. Representative immunofluorescent microscopy images of DARPP-32 (green color) and DAPI (blue color) staining of the striatum from vehicle (**A**), laquinimod-treated (**B**–**E**) R6/2 animals ((vehicle: n = 4, 0.5 mg: n = 5, 1.5 mg: n = 7, 5 mg: n = 8, 25 mg: n = 9) or WT littermates (**F**) (n = 6). Note the double stained cells exemplary marked with arrows. Bar = 50 μm. Quantification (**G**) revealed significant differences in the striatum after 5 mg/kg (p* < 0.05) and 25 mg/kg (p*** < 0.001) laquinimod treatment.

Previous studies demonstrated a downregulation of dopamine- and cAMP-regulated phosphoprotein, 32 KDa (DARPP-32) in the striatum of untreated R6/1 HD mice beginning at the age of five months and, especially, at 11 months of age^12, 33^. Here, we could detect significantly increased numbers of DARPP-32 positive cells in the striatum after laquinimod treatment (5 mg: n = 8, p* < 0.05; 25 mg: n = 9, p*** < 0.001) (Fig. 4).

### Laquinimod and BDNF expression

**Figure 5:**
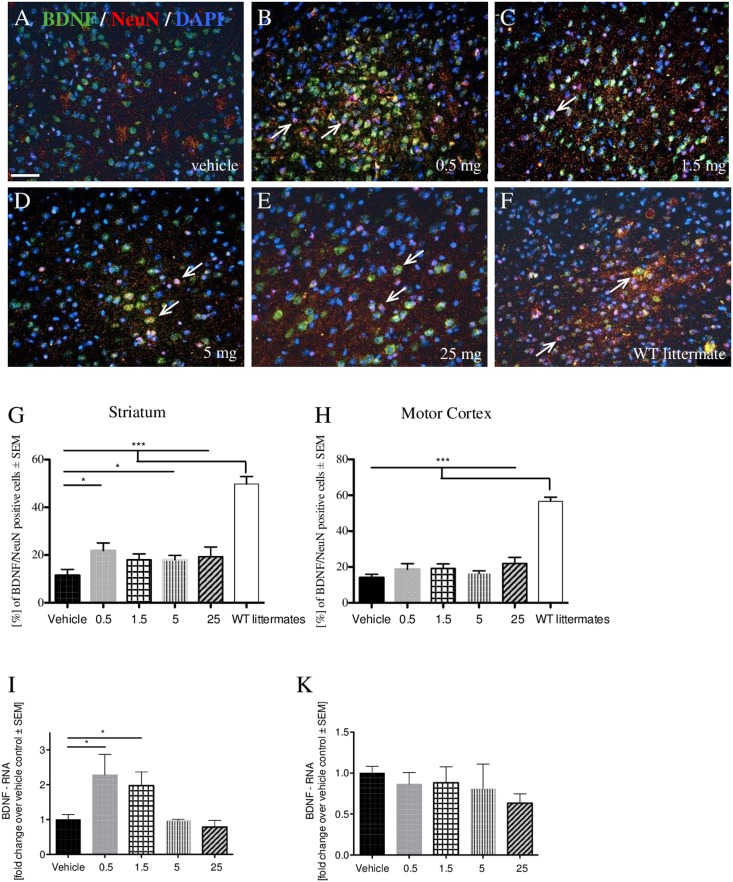
Laquinimod changes BDNF-levels. Representative immunofluorescent images of BDNF (green color), NeuN (red color) and DAPI (blue color) staining (**A**–**F**) (vehicle: n = 4, 0.5 mg: n = 5, 1.5 mg: n = 7, 5 mg: n = 7, 25 mg: n = 9, WT littermates: n = 6). Double stained neurons are marked with arrows. Bar = 50 μm. Blinded quantification of the striatum (**G**) and motor cortex (**H**) only showed a clear increase of BDNF-NeuN-double stained striatal neurons after treatment with 0.5 and 5 mg/kg laquinimod (p* < 0.05). mRNA levels in striatal (**I**) and cortical (**K**) tissues from 12-week-old vehicle and laquinimod-treated mice were analyzed: Both low concentrations (0.5 and 1.5 mg/kg) led to significantly higher scores compared to vehicle in the striatum (p* < 0.05) (**I**).

Immunofluorescence double staining for BDNF and NeuN in R6/2 brain tissue (Fig. 5A–F) exhibited yellow-pink colored cells when merged. Dosages of 0.5 and 5 mg/kg laquinimod led to a significant increase in BDNF-NeuN-co-expressing neurons only in the striatum of R6/2 transgenic mice (p* < 0.05) (Fig. 5G).

To further examine the effect of laquinimod on tissue-specific BDNF levels, we performed a quantitative rt-PCR of mRNA in the different groups (Fig. 5I,K).

Striatum BDNF mRNA expression further confirmed the immunohistochemical results: the amount of BDNF significantly (p* < 0.05) increased after treatment with 0.5 mg/kg laquinimod (Fig. 5I). Instead of BDNF/NeuN staining, the tissue of mice treated with 1.5 mg/kg (and not 5 mg/kg) showed a significant increase of BDNF versus vehicle (p* < 0.05). No significant changes in BDNF mRNA expression were seen in the motor cortex again (Fig. 5K).

### Laquinimod and its metabolic effects on Ecdysone inducible PC12 cell line model of HD

**Figure 6:**
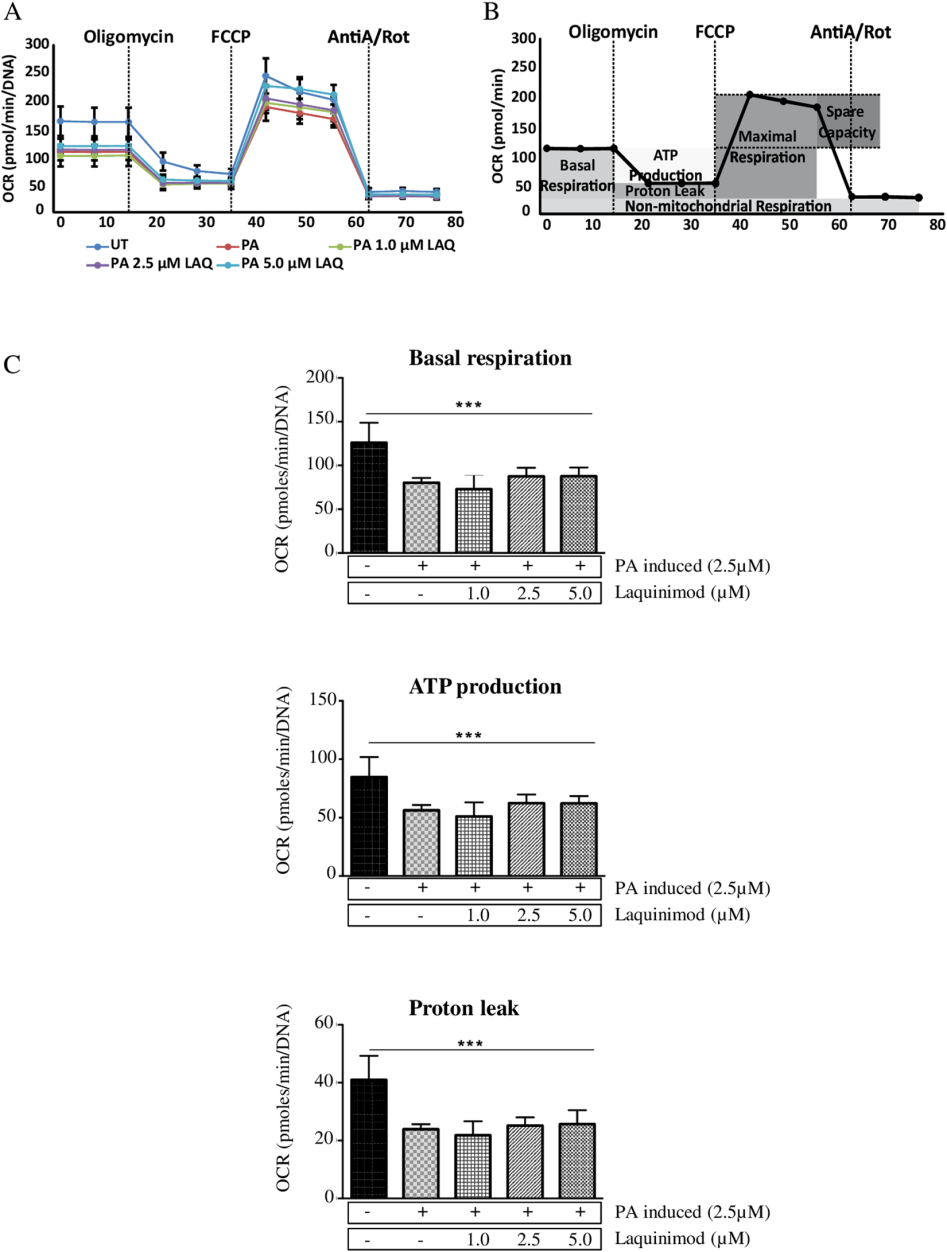
Laquinimod does not influence mitochondrial function *in vitro*. Oxygen consumption rates (OCR) were measured in PC12 cells expressing inducible exon 1 fragment of HTT gene with 103 glutamine repeats fused to enhanced green fluorescent protein 48 h after ponasterone A treatment (PA, 2.5 µM) and 0, 1.0, 2.5, or 5.0 µM laquinimod. (**A**) Basal respiration was measured at three time points following sequential injection of oligomycin (1.2 µM), FCCP (0.5 µM) and antimycin A/rotenone (1.0 µM each). (**B**) Representative OCR profile for calculation of key parameters of mitochondrial respiration shown in **C**. Basal respiration represents bioenergetic demands under baseline conditions. Proton leak is defined as basal respiration not coupled to ATP production. ATP production is determined by OCR decrease upon ATP synthase inhibition (**C**). Basal respiration, ATP production, and proton leak are decreased in mhtt-expressing PC12 cells but not influenced by laquinimod treatment. Data are represented as the mean ± SD.

Mitochondrial dysfunction and oxidative damage have been implicated in HD pathogenesis^34^. We used label-free, real-time measurement of the mitochondrial oxygen consumption rate (OCR), utilizing the Seahorse XF96 extracellular flux analyzer (Agilent Technologies). We measured the OCR in response to sequentially added oligomycin, FCCP, and antimycin A/rotenone in ponasterone A-induced PC12-mhtt-exon 1–103QP cells treated with laquinimod for 48 h at different concentrations (Fig. 6A). Subsequently, mitochondrial key parameters, such as basal respiration, ATP production, and proton leak (Fig. 6C), were calculated according to the exemplary respiratory profile shown in Fig. 6B. In line with published data, we observed reduced basal respiration and ATP production in PC12 cells expressing mhtt (Fig. 6A,B). However, the proton leak was also decreased upon mhtt expression, suggesting that a decrease in mitochondrial mass is responsible for the observed effects. Treatment of cells with laquinimod did not affect the impaired mitochondrial respiration in any concentration tested (Fig. 6C).

## Discussion

In this study, we showed that laquinimod exerted beneficial effects on motor functions and some features of neurodegeneration in R6/2 HD transgenic mice. This is in line with earlier studies using the YAC128 mice, and another HD mouse model also describing behavioral and motor improvement as well as reduced pathology in the striatum and certain cortical regions. Also, parallel to this study, no effect on body weight or survival was observed in either YAC128 or in our R6/2 mice.

We decided to assess laquinimod as its influence on BDNF may be relevant for its potential on mediating neuroprotection in HD.

BDNF is known to be a key player in the pathogenesis of HD but there are conflicting studies about underlying mechanisms and signaling pathways.

Several studies revealed a reduced level^18^ or even lack of BDNF^10^ in the striatum. In these studies, a reduced striatal BDNF mRNA level seems to correlate with reduced cortical BDNF mRNA expression. This implicates, if cortical BDNF mRNA expression is impaired this results in reduced BDNF supply to the striatum^12^.

Other studies state that there is a normal striatal BDNF level at early and intermediate disease stages of HD^35–38^ and even late disease stages with normal levels were described^35–37, 39, 40^. This is supported by normal cortical BDNF expression in symptomatic BACHD mice^41^ and human HD tissue samples^42, 43^. This suggests that possibly further downstream defects may underlie these histopathological deficiencies.

BDNF-TrkB signaling pathway is discussed to be a basic mechanism^12–14, 44^. Expression of TrkB receptor by MSNs is relevant for BDNF^12^. The cited studies assume that MSNs, 95% of them located in the striatum, do not produce BDNF by themselves. Instead, they are dependent on BDNF-TrkB signaling pathway. At the same time, striatal cellular processes are sensitive to mhtt and this is linked to nuclear factor kappa B (NFκB) activity^45^. NFκB signaling is enhanced in R6/2 mice^46^ and additionally mhtt causes increased NFκB activity^45, 47^. Interaction of both pathways may explain effects of laquinimod as laquinimod reduces NFκB activation in astrocytes and restores BDNF levels^28, 48, 49^.

Our pronounced increase of BDNF is difficult to explain. Maybe the increase of mRNA levels in the striatum and not in the motor cortex after laquinimod treatment can be explained by conditions of general, cortical and striatal reduction of BDNF levels before treatment. Modulation of NFκB activity might restore BDNF levels especially in the striatum, since this area is the most affected in HD by mhtt with high influence of NFκB pathway-effects. In short, laquinimod may act at the most important region of interest. Taken together, results of the different studies including our results remain conflicting and further studies are needed to elucidate the different pathways and mechanisms.

We performed histochemical analyses to elucidate whether laquinimod has a neuroprotective capacity. Increased numbers of DARPP-32 positive cells, as well as reduced levels of EM48- and ubiquitin-positive cells in the striatum, underline the protective effects on neuronal function. These results are in line with further studies that declare a significant neuronal loss and striatal atrophy in R6/2 mice without therapeutic intervention^12, 30, 31, 50^. These changes were only seen in the striatum and not in the motor cortex as BDNF signaling is known to induce expression of DARPP-32, a striatal-enriched protein, that by itself is essential for striatal MSN function^51–53^. Once again, the differences in the striatum and motor cortex might be explained by the positive influence of BDNF, especially on striatal cells, as mentioned above. Data of BDNF-NeuN double staining underline this hypothesis.

Most effects in our study were observed in the striatum. However, represented by mhtt- and iNOS staining, there were positive effects not only in the striatum but also in the motor cortex area. Remarkably, mhtt was reduced after laquinimod treatment. The potential mechanism linking laquinimod to mhtt aggregation cannot yet be explained. Hypothetically, NFκB pathway signaling, among others, plays a potential role. It is known that laquinimod increases IκB expression and decreases NFκB gene expression^47, 54, 55^. At the same time, mhtt interacts with IκB and upregulates NFκB gene expression. Perhaps laquinimod prevents the over-activation of NFκB signaling and reduces immune response. Mhtt protein folding might be reduced by lower levels of immunological factors and lower cell stress.

To date, this is merely hypothetical, and further studies are warranted. Data after iNOS staining are in line. Up-regulation of NFκB signaling results in increased production of pro-inflammatory mediators, represented by iNOS^55^, and this effect is prevented by laquinimod. Mechanisms cannot yet be proved. Detailed quantification of activated microglia, examination of more specific microglial markers, and analysis of laquinimod effects on NFκB signaling pathway are planned for future projects. In summary, we assume additional neuroprotective mechanisms by laquinimod not only based on BDNF. Immunomodulative effects, reduced caspase-6 activation, and interaction with iNOS were, among others, already described as potential mechanisms of action in laquinimod^27, 28, 56^.

NO dysfunction in CNS has been previously highlighted to advance progressive striatal damage in both HD animal models and HD patients^57, 58^. So far, both increased iNOS expression^59, 60^ and a decrease in activity and expression have been observed in HD transgenic mice^57, 58^. In our study, we observed a reduction of iNOS positive cells after treatment in both the striatum and motor cortex. We hypothesized that the positive effect of laquinimod on NO is, among other things, based on the reduction of oxidative stress. This hypothesis could not be certified by our results of metabolic activities in PC12 cells. We could not detect changes in ATP production and basal respiration after treatment with laquinimod. The reasons for this could be twofold. On the one hand, to some degree, there might be a model effect. The electron transport system^2^ consistent with the respiratory complexes I-IV is utilized mainly to generate ATP. Defects in the ETS are differently expressed, depending on the expression of N-terminal fragments or full-length models of HD^61–63^. On the other hand, other already discussed mechanisms that have not been focused in this study, such as immunomodulative effects or reduced caspase-6 activation, might be responsible for general neuroprotective effects after laquinimod^27, 28, 56^. This might be a reason for the reduced mhtt levels that we have seen in our study. Immunomodulative effects under laquinimod are a very well-known mechanism of action and have already been discussed in many other studies^26, 64–66^ aimed at inflammatory as well as neurodegenerative diseases, including HD^29^. As the immunomodulative effects of laquinimod in YAC128 with reduced interleukin 6 levels were already confirmed^29^, we did not focus on this topic; neither did we re-analyze caspase-6 pathways.

In summary, our findings suggest that treatment with laquinimod could provide a potential mild neuroprotective effect in HD mice, which is consistent with a previous study of YAC128. Besides the influences of laquinimod on the immune system, our work strengthened the effect of laquinimod on BDNF-pathways in HD.

Given its side effect profile, and suggesting that long-term treatment is well-tolerated, laquinimod may be a potential disease-modifying agent for the treatment of HD and other neurodegenerative diseases. Clinical trials in HD have recently started and the first results are anticipated soon.

## Materials and Methods

### Animal models and treatment procedure

Heterozygous R6/2 ovarian transplanted (OT) female mice with 160 CAG repeat length were purchased from Jackson Laboratory (Stock: 002810, Bar Harbor, ME, USA). R6/2 mice were obtained by breeding OT-R6/2 female mice with wild-type male mice from their background strain (OT-R6/2: B6CBAF1/J). Transgenic offspring were identified by PCR genotyping using tail-tip samples. Stability of CAG repeat size was verified (160 ± 5 repeats) for each sample. All experiments were conducted on F1 and F2 transgenic mice to limit CAG repeat variability. Only transgenic heterozygous females were included in the studies to avoid gender-related differences, and treatment started between 30 and 32 days of age.

All experiments and protocols were approved by the North-Rhine-Westphalia authorities for animal experimentation (LANUV, approval ID: 84-02.04.2011.A400, §8 Protection of Animals Act), and all methods were carried out in accordance with relevant guidelines and regulations.

Mice were randomly divided into four treatment groups and two control groups. Groups were matched for repeat length. They received either laquinimod in different concentrations of 0.5 mg/kg body weight (bw) (n = 23), 1.5 mg/kg (n = 26), 5 mg/kg (n = 26), and 25 mg/kg (n = 25), or water (n = 16), as well as WT littermates as control (n = 16). Laquinimod (ABR-215062) was provided by Teva Pharmaceutical Industries, Ltd (TEVA Pharmaceutical Companies). The substance was dissolved in water and given by daily oral gavage at a total volume of 200 µl. Mice were monitored for clinical condition daily, and weight was scored three times a week. For survival analysis, treatment continued throughout the experiment until mice either died or became moribund.

### Behavioral analyses

For behavioral studies, motor coordination and balance were tested for all animals on a five-station mouse rotarod (Ugo Basile, Biological Research Apparatus, Varese, Italy) every other week starting from week four of age. Mice were first trained twice at the age of three weeks on the rotarod at a constant speed of 10 rpm with a maximum of 240 seconds (s). Subsequently, during test sessions, the speed of rotation was increased from 4 to 40 rpm over a period of 240 s. Mice were given two trials on the rotarod on the same day. Their latencies to fall were measured and averaged.

### Histological and immunohistochemical analyses

On day 85 (±2 days) animals were perfused with 4% paraformaldehyde in phosphate buffered saline (PBS, pH 7.4) under deep anesthesia with ketamine/xylazin/acepromazin (65 mg/20 mg/3 mg/kg). For brain histology and immunohistochemistry, we used 5 µm paraffin-embedded sections (Bregma 0.14 mm to Bregma 1.10 mm). To assess neuronal degeneration, cresyl violet staining was performed. The histological procedures essentially followed previously described protocols^67, 68^.

Nitric oxide synthase (NOS) activity and NOS expression were measured labeling its subtype inducible NOS (iNOS, 1:50, Enzo, Switzerland). Mutant htt aggregates were detected by using a mouse anti-ubiquitin antibody (1:1000; Chemicon, Temecula, USA) or anti-huntingtin protein (clone mEM48, Millipore, Cat. #MAB5374; 1:500). After blocking with a 10% bovine serum albumin (BSA) in PBS for 30 minutes, sections were incubated with the first antibody in 1% BSA in phosphate-buffered saline (PBS) overnight at 4 °C. Hence, sections were washed three times with PBS, followed by an incubation with the second antibody (biotinylated goat anti-mouse or anti-rabbit IgG) in 1% BSA in PBS for 45 minutes. After incubation with the avidin-biotin-complex (Vectastain, Vector Laboratories, Wertheim, Germany), reaction was developed with diaminobenzidine tetrahydrochloride (Merck, Darmstadt, Germany).

For immunofluorescence staining of MSNs marker dopamine- and cAMP-regulated phosphoprotein = DARPP-32 (DARPP-32 1:100, Abcam, England), sections were boiled in citrate buffer for 25 minutes and then blocked with 5% horse serum and 0.25% Triton X in TBS for 60 minutes. Next, the primary antibody, diluted in 1% horse serum and 0.25% Triton X100 in TBS, incubated overnight at 4 °C. The second antibodies, Alexa 488 and Alexa 555 (1:1000, Invitrogen, Frankfurt, Germany *later* Lifetechnologies Carlsbad, California/USA), as well as DAPI (1:1000), were diluted in 1% horse serum and 0.25% Triton X100 in TBS, and incubated for one hour.

The same protocol was used for double immunofluorescent staining of BDNF (BDNF 1:100, Millipore, Temecula, USA) and the neuronal marker NeuN (NeuN 1:200, Chemicon, Temecula, USA). At first, sections were stained for BDNF with a primary antibody incubation time of 48 hours. After that, the staining for NeuN was performed separately. Stained sections were mounted with ProLongGold. Quantification was performed in a blinded manner for all stainings.

For light microscopy (BX51 Olympus, Hamburg, Germany) fixed medial, lateral, ventral, and rostral areas in the striatum and motor cortex M1 and M2 layers were evaluated (Bregma 0.14 mm-1.10 mm). Fluorescent analyses were performed utilizing the inverted fluorescence microscope (BX51; Olympus, Tokyo, Japan) equipped with an Olympus DP50 digital camera.

### Real-time PCR analysis

Brains were collected after perfusing with 0.9% saline transcardially, and motor cortex and striatum were dissected and homogenized in 1 ml of trizol (Invitrogen, Darmstadt, Germany) using a pellet pestle homogenizer motor. Snap freezing in liquid nitrogen and storage at −80 °C until use followed. RNA was isolated with the Qiagen RNA Lipid Isolation Kit according to the manufacturer’s protocol. After the extraction, cDNA was amplified by reverse transcription using SuperScript II Reverse Transcriptase (Invitrogen, Darmstadt, Germany). Real-time PCR was performed with SYBR Green GoTaq^®^ qPCR Master Mix (Promega, France) on an Applied Biosystems 7200 Real-time PCR System (Applied Biosystems, Darmstadt, Germany). BDNF mRNA expression was normalized to the geometric average expression of two reference genes (ß-actin and GAPDH). Declared fold change is relative to vehicle as control, n = 4 per group. Reactions were performed in triplicates for each cDNA sample in a 96-well plate. Primers were as follows: mouse BDNF (forward: 5′-TGC AGG GGC ATA GAC AAA AGG-3′; reverse: 5′-CTT ATG AAT CGC CAG CCA ATT CTC-3′); mouse β-actin (forward: 5′-CAT GTT TGA GAC CTT CAA CAC CCC-3′; reverse: 5′-GCC ATC TCC TGC TCG AAG TCT AG-3′); and mouse GAPDH (forward: 5′-ACG ACC CCT TCA TTG ACCTC-3′; reverse: 5′-GGG GGC TAA GCA GTT GGT GG-3′).

### Seahorse Experiments

Here, we utilized an ecdysone-inducible rat pheochromocytoma (PC)12 cell-line model of HD expressing the exon 1 fragment of HTT gene with 103 glutamine repeats fused to EGFP (PC12-mhtt-exon 1-103QP-EGFP)^32^.

This cell line was kindly gifted by E. Kloster (Ruhr University, Bochum, Germany) and was maintained at 37 °C in a humified 5% CO_2_ and 95% air atmosphere in the growth medium (Dulbecco’s modified Eagle’s medium, DMEM) (Sigma Aldrich) supplemented with 10% heat-inactivated fetal bovine serum (Sigma-Aldrich) and 1% penicillin/streptomycin (Gibco). For Seahorse, metabolic activity assay cells were seeded on poly-D-lysine coated sterile 96-well plates at a density of 7.5 × 104 cells per well. Following induction with 2.5 µM Ponasterone A (Sigma-Aldrich) for transgene expression and subsequent incubation with laquinimod (1 µmol/l, 2.5 µmol/l and 5 µmol/l) for 48 hours, we measured the metabolic activities of the cells using a Seahorse Bioscience XF96 Extracellular Flux Analyzer (North Billerica, MA. USA). The concentrations of laquinimod used in this experiment correspond to the oral 0.6 mg, 1.5 mg and 3 mg used in laquinimod clinical trials and previously reported *in vitro* experiment with laquinimod^65, 69^. For the Seahorse metabolic activity measurement, we used DMEM (Sigma-Aldrich) as a medium supplemented with 2 mM sodium pyruvate (Sigma-Aldrich), 10 mM glucose (Sigma-Aldrich), and 2 mM L-glutamine (Gibco^®^, ThermoFisher Scientific, USA) and BOFA mitochondrial function assay, as described by Seahorse Bioscience. We utilized sequential injections of Oligomycin (1.2 µM) as ATP synthase inhibitor, FCCP (0.5 µM) as potent mitochondrial oxidative phosphorylation uncoupler, and Antimycin A/Rotenone (1.0 µM) (Antimycin A: complex III inhibitor and Rotenone: mitochondrial electron transport chain complex I inhibitor and potent NADH oxidation inhibitor (Seahorse Bioscience). All cell culture experiments were performed at ambient (21%) O_2_. Post-measurement, the data were normalized using CyQUANT^®^ Cell Proliferation Assay according to the manufacturer’s instructions. Key parameters of mitochondrial function, such as basal respiration, ATP-linked respiration, proton leak, maximal respiration, and spare respiratory capacity, were analyzed after laquinimod treatment. These parameters were determined using the included Seahorse analysis software. Results were compared to untreated-uninduced condition and untreated-induced condition.

### Quantification and statistical analyses

Histochemical and immunohistochemical analyses were performed completely blinded on standardized 2 mm sections that were prepared using a brain slicer (Bregma 0.14 mm–1.10 mm) in eight fixed regions (4 motor cortex: M1 layer ventral and rostral area on both sides 4 striatum: medial and lateral striatum) under 20 respectively 40-fold magnification. Two microscope slides per animal with two preparation cuts on each slide were analyzed for each staining. Counting was done with ImageJ32 software (W. Rasband, National Institutes of health, USA). Data are provided as a means of counting result/number of positive cells in analyzed fields of view ± SEM. For histological/immunohistochemical evaluations, statistical analysis was performed by ONE-Way-ANOVA of the total number of animals used with Bonferroni post hoc test. To assess the survival of mice, a Kaplan-Meier analysis with a log rank test was used (all analyses were done by Graph Pad Prism 5, San Diego, CA, USA). A probability level of p* < 0.05, p** < 0.01, p*** < 0.001 was considered to be statistically significant for all tests.
