# Author Correction: Laquinimod treatment in the R6/2 mouse model

**DOI:** 10.1038/s41598-018-37926-4

**Published:** 2019-03-15

**Authors:** Gisa Ellrichmann, Alina Blusch, Oluwaseun Fatoba, Janine Brunner, Christiane Reick, Liat Hayardeny, Michael Hayden, Dominik Sehr, Konstanze F. Winklhofer, Carsten Saft, Ralf Gold

**Affiliations:** 1 Department of Neurology, St. Josef-Hospital, Ruhr-University Bochum, Bochum, Germany; 2 Center of Clinical Research, Ruhr-University Bochum, Bochum, Germany; 3 Galmed Pharmaceuticals, Tel Aviv, Israel; 4 Teva Pharmaceutical Industries Ltd, Tiqva, Israel; 5 Department of Molecular Cell Biology, Institute of Biochemistry and Pathobiochemistry, Ruhr-University Bochum, Bochum, Germany

Correction to: *Scientific Reports* 10.1038/s41598-017-04990-1, published online 10 July 2017

Christiane Reick was omitted from the author list in the original version of this Article. This has been corrected in the PDF and HTML versions of the Article.

The Author Contributions section now reads:

G.E. designed the study, carried out the experiments, analyzed and interpreted results, performed statistics and drafted the manuscript. A.B. supported experiments and histology/immunohistochemistry. O.W. supported histology, immunohistochemistry, analysis of results and seahorse experiments. J.B. supported experiments. C.R. supported histology, immunohistochemistry and analysis of results L.H. and M.H. provided general support. D.S. carried out the seahorse experiments. K.F.W. provided general support and inspected the manuscript. C.S. participated in study design and supported drafting the manuscript as well as revision data. R.G. provided general support, participated in the design of the study and inspected the manuscript.

